# Advances in Nickel-Containing High-Entropy Alloys: From Fundamentals to Additive Manufacturing

**DOI:** 10.3390/ma17153826

**Published:** 2024-08-02

**Authors:** Ashish Kumar Gupta, Amit Choudhari, Aditya Rane, Abhishek Tiwari, Prince Sharma, Ashutosh Gupta, Prathamesh Sapale, Ravi Teja A. Tirumala, Rajmohan Muthaiah, Abhishek Kumar

**Affiliations:** 1 School of Mechanical and Aerospace Engineering, Oklahoma State University, Stillwater, OK 74078, USA; aditya.rane10@okstate.edu; 2 Department of Mechanical Engineering, Cleveland State University, Cleveland, OH 44115, USA; a.choudhari@vikes.csuohio.edu; 3 Department of Mechanical and Aerospace Engineering, Indian Institute of Technology Hyderabad, Sangareddy 502284, India; 4 Department of Mechanical Engineering and Mechanics, Lehigh University, Bethlehem, PA 18015, USA; prs221@lehigh.edu; 5 Department of Zoology, Dayanand Vedic College, Orai 285001, India; mailboxakg22@gmail.com; 6 School of Chemical Engineering, Oklahoma State University, Stillwater, OK 74078, USA; prathamesh.sapale@okstate.edu (P.S.); raddank@okstate.edu (R.T.A.T.); 7 School of Aerospace and Mechanical Engineering, University of Oklahoma, Norman, OK 73019, USA; rajumenr@ou.edu; 8 J. Mike Walker ’66 Department of Mechanical Engineering, Texas A&M University, College Station, TX 77840, USA; akumar71@tamu.edu

**Keywords:** high entropy alloys, multi component alloys, Ni alloys, hardness, additive manufacturing, oxidation, corrosion

## Abstract

High-entropy alloys (HEAs) are recognized as a class of advanced materials with outstanding mechanical properties and corrosion resistance. Among these, nickel-based HEAs stand out for their impressive strength, ductility, and oxidation resistance. This review delves into the latest advancements in nickel-containing HEAs, covering their fundamental principles, alloy design strategies, and additive manufacturing techniques. We start by introducing HEAs and their unique properties, emphasizing the crucial role of nickel. This review examines the complex relationships between alloy composition, valence electron concentration (VEC), and the resulting crystal structures. This provides insights into design principles for achieving desired microstructures and mechanical properties. Additive manufacturing (AM) techniques like selective laser melting (SLM), electron beam melting (EBM), and laser metal deposition (LMD) are highlighted as powerful methods for fabricating intricate HEA components. The review addresses the challenges of AM processes, such as porosity, fusion defects, and anisotropic mechanical properties, and discusses strategies to mitigate these issues through process optimization and improved powder quality. The mechanical behavior of AM-processed nickel-based HEAs is thoroughly analyzed, focusing on compressive strength, hardness, and ductility. This review underscores the importance of microstructural features, including grain size, phase composition, and deformation mechanisms, in determining the mechanical performance of these alloys. Additionally, the influence of post-processing techniques, such as heat treatment and hot isostatic pressing (HIP) on enhancing mechanical properties is explored. This review also examines the oxidation behavior of nickel-containing HEAs, particularly the formation of protective oxide scales and their dependence on aluminum content. The interplay between composition, VEC, and oxidation resistance is discussed, offering valuable insights for designing corrosion resistant HEAs. Finally, this review outlines the potential applications of nickel-based HEAs in industries such as aerospace, automotive, and energy, and identifies future research directions to address challenges and fully realize the potential of these advanced materials.

## 1. Introduction

Metals account for roughly two-thirds of all elements on Earth and approximately 25% of Earth’s mass [1,2]. Metals are generally not used in their pure form because they fail to exhibit good mechanical, structural, and thermal properties unless alloyed [3,4]. This property limitation is overcome by alloying the pure metal with other elements. Traditional alloys provide basic essential features by adding basic essential elements, such as iron, titanium, or aluminum, and adding minor amounts of other factors to regulate the microstructure and enhance the characteristics [5,6]. For hundreds of years, this alloying method has been routinely employed. The quantity of potential compositions limits the traditional design of new alloys [7]. A novel and advanced class of alloys known as multi-principal element or high entropy alloys (HEA) are synthesized by mixing more than four elements in near equal ratios. Depending on the constituting elements, these alloys provide exceptional hardness, high yield stress, and enhanced corrosion resistance, plasticity, and thermal conductivity [8,9,10,11,12,13,14,15,16]. HEAs were first reported in 2004 individually by Yeh and Cantor [17,18]. The nano-structured fine-grained materials (grain size 0.1 µm) have garnered overwhelming interest as contrary to their coarse-grained counterpart [19,20,21]. The exceptional properties of the nano-structured materials originate from higher density grain boundaries, statistically increasing the defect density, hindering the dislocation motion and enhancing corrosion protection [22,23,24]. A widely used synthesis technique for nano-structured materials is ball/mechanical alloying (powder metallurgy), which usually activates a material to nearly 30 kJ/mol [25] followed by consolidation using sintering techniques such as spark plasma sintering. This process is compromised further by the thermal stability of the nano-structured grains as they tend to coarsen under working pressure–temperature conditions. Experiments with low melting nanocrystalline Sn, Cu, Al, Mg, and Pb have shown grain coarsening even at room temperature [26,27]. Moreover, it is technically challenging to counter the trade-off between grain coarsening in alloy powder during the sintering process [28]. It was further revealed that the properties of nano-structured alloys with 1–2 alloying elements did not improve mechanical performance much. Hence, the idea of high configurational entropy was leveraged to stabilize these nano-structured materials as incorporated within high entropy alloys.

Franz Carl Achard, a German physicist and metallurgist, studied alloys that had 5–7 components in equal quantities in the late 18th century, and is regarded as one of the pioneers of HEA research [29]. He studied more than 900 possible alloy combinations made from the metals that were known at the time, such as silver, cobalt, iron, lead, zinc, bismuth, copper, tin, antimony, arsenic, and platinum, and the results were published in his book. Despite the fact that HEAs encompass a broad spectrum of principal elements, they consistently exhibit simple crystal structures. These include body-centered cubic (BCC) [30], face-centered cubic (FCC) [18,31], and hexagonal close-packed structures [32,33], or a combination of these structures [17].

The intriguing characteristics of HEAs significantly enhance their appeal. For example, FCC-structured CrCoNi HEAs exhibit exceptionally high fracture toughness, rivaling cryogenic steels; superior fatigue resistant, oxidation, and magnetic properties; and remarkable irradiation resistance [34,35,36,37,38,39]. 

The stabilization of HEAs is anchored to four primary effects [40]: (a) high configurational entropy; (b) severe lattice distortion; (c) sluggish diffusion; and (d) cocktail effect [41,42]. While it is frequently demonstrated that large configurational entropy (ΔS_mix_ ≥ 1.5R, R being the universal gas constant) serves as a stabilizing factor for equiatomic multicomponent HEAs [17,43,44], this might not always be accurate due to the interplay between enthalpy and entropy [45,46].

Nickel-based super alloys have been used as high-temperature load-bearing candidates for the past seven decades because they can withstand temperatures up to 1100 °C. They possess excellent room temperature ductility and good creep and fatigue behavior at relatively high temperatures. In the same direction, Ni-based HEAs are developed to further increase the limits of Ni superalloys. This review outlines the potential applications of nickel-based HEAs in industries such as aerospace, automotive, and energy, and identifies future research directions to address challenges and fully realize the potential of these advanced materials.

## 2. High Entropy Alloys (HEAs)

HEAs consist of multiple principal elements, often four or more, in near-equimolar ratios, along with minor elements. The core concept of HEAs is that solid solution phases exhibit higher stability due to significantly increased mixing entropy compared with intermetallic compounds, especially at elevated temperatures. This makes HEAs suitable for efficient synthesis, processing, analysis, and property tuning [38].

Do complex concentrated alloys, or multicomponent alloys, come under the umbrella of HEAs? Considering an equimolar solid solution, based on Boltzmann’s hypothesis on the relationship between entropy and permutations of all possible states, R ln(n) can be used to calculate its configurational entropy per mole (Equation (1)):

(1) $$∆S_{conf}=-k\ln w=-R\ln\frac{1}{n}=R\ln n$$

R is gas constant, 8.314 J/K mol, and n is the number of elements.

Physically, entropy has four contributors, namely, configurational, vibrational, magnetic, and electronic. Configurational entropy dominates the other three at room temperature, as compared with vibration at high temperatures [47]. Vibrational entropy at high temperatures could be calculated from phonon calculations in the framework of density functional theory [48,49]. Excess vibrational entropy is found to takeover configurational entropy and lead to phase stabilization at high temperatures [50]. Vibrational entropy is dependent upon mass disorder in the solid solution, which again originates from the incorporation of different elements in alloys [12,49].

In Table 1, the configurational entropy of a three-component equimolar solution exceeds the threshold of 1R, and that of a five-component solid solution is 61% greater. Consequently, it is plausible to consider that 1.5R (even without considering the other three mixing entropy contributions mentioned above) is sufficient to counterbalance mixing enthalpy and can be used as a threshold between HEAs and medium-entropy alloys [47]. This implies that only a select few conventional alloys possess a high configurational entropy greater than 1.5R. The configurational entropy equation indicates that when an element is present at a concentration of 5%, it contributes a mixing entropy of 0.05Rln0.05 equivalent to 0.15R. However, this amount constitutes only 10% of the minimum threshold of 1.5R. Therefore, an element present in a quantity exceeding 5 at.% could be classified as a primary element. The contributions for 4 at.%, 3 at.%, 2 at.%, and 1 at.% are 0.129R, 0.105R, 0.046R, and 0.078R, respectively, corresponding to percentages of 8.6%, 7%, 5.2%, and 3.1% based on 1.5R. Consequently, we categorize an element in a quantity of 5 at.% as a minor element. The question about HEAs’ metallic principal elements’ upper limit arises due to the data in Table 1 and Equation (1). The total configurational entropies for 6, 9, and 11 element alloys, along with 13, 14, 15, 20, and 40 element equal-mole alloys, are 1.79R, 2.2R, 2.4R, 2.57R, 2.64R, 2.71R, 3.0R, and 3.69R, respectively. The change of 0.07R from a 13 element to a 14 element alloy is small (0.07/2.57 = 2.7%), leading to a suggestion of a practical range of principal elements between 5 and 13 for HEAs.

From a chosen set of 13 metallic elements, one can create 7099 different combinations for designing equal-mole HEA systems. This encompasses systems with 5 to 13 elements, adhering to the practical limit of 13 metallic elements as defined earlier [21].

$$C_{5}^{13}+C_{6}^{13}+C_{7}^{13}+C_{8}^{13}+C_{9}^{13}+C_{10}^{13}+C_{11}^{13}+C_{12}^{13}+C_{13}^{13}=7099$$

We could develop an equimolar AlCoCrCuFeNi alloy. We can also create unequal-mole alloys based on structure property relation, with minor alloying elements like AlCo_0.5_CrCu Fe_1.5_Ni_1.2_B_0.1_C_0.15_ for further microstructure and property modification. As a result, there are an infinite number of HEAs [47,51]. Table 2 Shows ∆S_conf_ of some commonly used alloys.

## 3. Selection of Elements for HEA

The existing literature and bars can be used to identify candidate major constituent elements for possible use in alloys. Table 3 shows possible candidates drawn from the periodic table, excluding radioactive elements. The selection of candidate elements is based on desired attributes such as temperature, density, and strength, among others. Operating temperatures are classified as Low-T (200 °C), Medium-T (600 °C), and High-T (1000 °C) [40,52]. Microstructural properties determine strength, whereas melting temperature is determined by constituents, atomic sizes, and chemical interactions. The Pauling electronegativity and atomic radius are essential factors in alloying element selection [32,44]. The use temperature must be 50% of the temperature at which the alloy will be used. The Hume–Rothery rule identifies individual elements with atomic radii differences within 8%, which is crucial for the synthesis of single-phase alloys [40,52].

Achieving a trade-off between strength and tensile ductility in single-phased HEAs is challenging [13,15,18,53,54,55]. Studies indicate that single-phased FCC-structured HEAs are ductile but lack sufficient strength [31,56,57,58]. HEAs also suffer from elemental segregation leading to composition heterogeneity, which negatively affects structural properties and limits engineering application [59]. Single-phased BCC-structured HEAs are found to be extremely hard, and hence are not ductile enough to be machined or formed [35].

**Figure 1:**
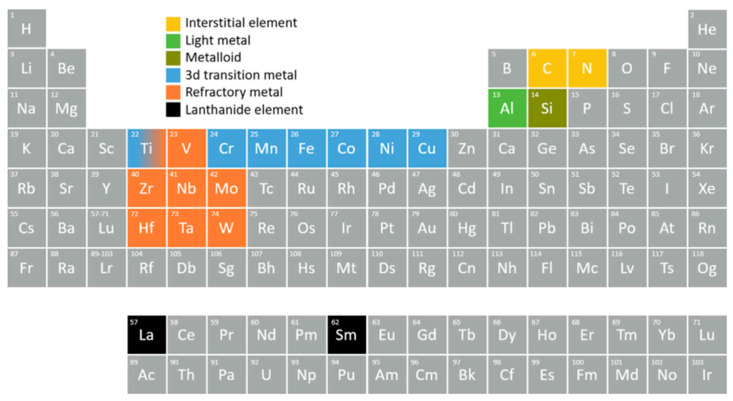
The primary components utilized for AM HEAs are highlighted by clusters of alloying elements [60]. Adapted with permission from ref. [60]. A 2022 Creative Commons Attribution (CC BY) license.

Figure 1 depicts a set of elements chosen to synthesize HEA components through additive manufacturing (AM) methods. These elements primarily include lightweight Al, 3D transition metals, refractory metals, the metalloid Si, and lanthanides La and Sm [60]. In certain variations of HEAs, additional substitutional or interstitial elements such as carbon and nitrogen are mixed as well.

## 4. Mechanical Properties and Defects of HEAs Processed by Additive Manufacturing

Hardness, tensile, and compression testing, among other AM-HEAs’ mechanical properties, have been investigated. Post-treatment processes like HIP, solution heat treatment, and aging can enhance performance by eliminating metallurgical defects and residual stress in the fabricated machine components [61,62].

### 4.1. Microhardness

**Figure 2:**
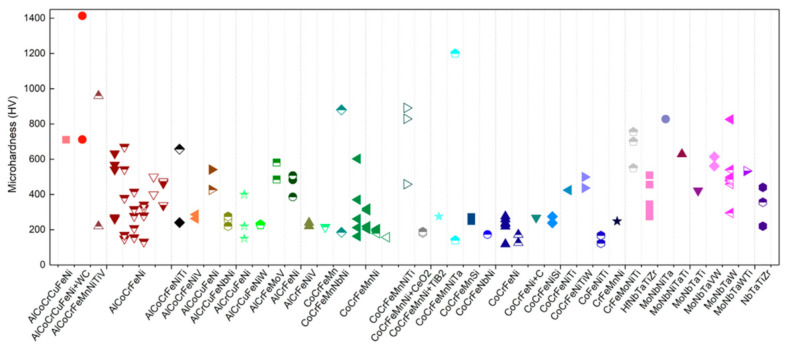
Data on the microhardness of AM HEAs were gathered from Zhang et al. [60]. (The HEAs produced by SLM, LMD, EBM, and WAAM are represented by the solid, top half-solid, open, and bottom half-solid symbols, respectively. Only considering the condition of fabrication). Adapted with permission from ref. [60]. A 2022 Creative Commons Attribution (CC BY) license.

A summary of the structural properties, including microhardness, and the printing parameters of HEAs processed by various additive manufacturing processes is presented in Table 4 and Table 5. In Figure 2, the hardness values of HEAs range from 100 to 1400 HV, influenced by the processing methods and elemental makeup of alloy. In general, HEAs with a BCC crystal structure (e.g., MoNbTaW) exhibit more complex dislocation motion compared with HEAs with an FCC phase (e.g., CoCrFeNi). The more considerable lattice distortion relative to the FCC phase originates from the enhanced solid solution strengthening of the BCC structure in HEAs [60].

### 4.2. Tensile Properties

**Figure 3:**
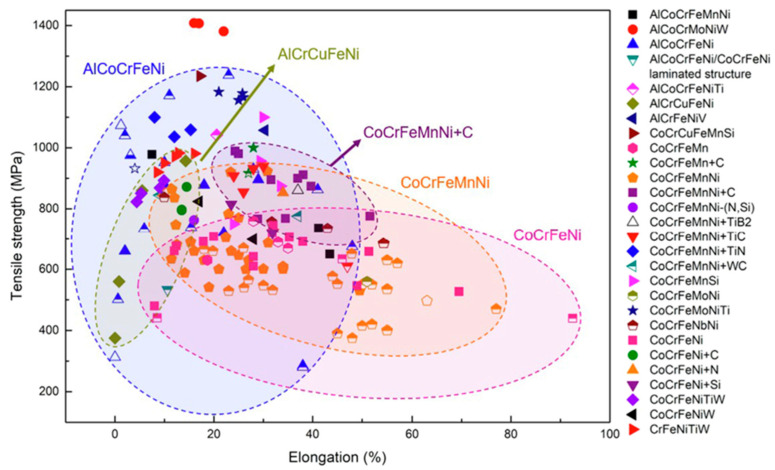
Different AMed HEAs’ UTSs against fracture elongation. The HEAs manufactured by SLM, LMD, EBM, and WAAM, respectively, are represented by the solid, top half-solid, open, and bottom half-solid symbols (just considering the as-fabricated state) [60]. Adapted with permission from ref. [60]. A 2022 Creative Commons Attribution (CC BY) license.

Ultimate tensile strengths (UTS) *v*/*s* elongation at fracture for several additively manufactured HEAs are presented in Figure 3. The UTSs of various HEAs range from 250 to 1400 MPa. It is evident that within a single HEA system, such as AlCoCrFeNi, the tensile characteristics varied significantly depending on the microstructure achieved using various AM processes and the selected processing parameters [63]. According to Xiang et al. [64] microstructure modification might lead to tuning the structural properties of CoCrFeMnNi specimens via altering the laser parameters such as power and scanning rates. It was reported that the dominating (001) pattern changed to a random texture as the laser power rose from 1 to 1.4 KW, the anisotropy decreased in the structure, and enhanced structural properties in contrast to the alloys were produced by melting-casting. In terms of the Hall–Petch relation, described in [65,66,67], it was discovered that additively made HEAs obey this relationship. The yield strength of a polycrystalline material is dependent on the statistical distribution size of the grains. The CoCrFeNi HEA deposited via SLM exhibits remarkable strength and ductility due to the lack of brittle intermetallic compounds or secondary phases. According to Guan et al. [68], the tensile deformation of the LMD-deposited CoCrFeMnNi HEA primarily involved dislocation actions aided by deformation twins, resulting in a tensile strength comparable to that of counterparts made from finer-grained wrought-annealed materials [60]. The UTS and fracture elongation of AM-processed HEAs demonstrate high variability even within the same alloy systems. This significant variance is attributed to the different microstructures obtained from the diverse AM technologies and processing parameters employed. For instance, in laser metal deposition (LMD)-fabricated CoCrFeMnNi, the tensile properties are tuned by varying the laser parameters such as power and scanning patter/rate which modifies the crystallographic texture [69].

**Table 4:** The mechanical properties (tensile properties and hardness) of various Ni-containing HEAs.

HEAs System	AM Techniques	Powder Size (μm)	Density	Relative Density	Printing Parameters	Phase Compositions	Yield Stength (MPa)	Ultimate Tensile Strength (MPa)	Elongation (%)	Hardness (HV)	Ref.
Al_0.3_CoCrFeNi	SLM	20–42	7.76	99.9	P = 150–170 W, v = 1100–1300 mm s^−1^, t = 25–30 µm, h = 45 µm	FCC	~730	896	29	-	[70]
Al_0.5_CoCrFeNi	SLM	15–45	-	-	P = 400 W, v = 270 mm s^−1^, t = 40 µm, h = 90 µm	FCC	579	721	22	262.5 ± 5	[71]
AlCoCrFeNi	EBM	45–105	-	-	-	FCC + BCC	-	-	-	-	[72]
Al_0.1_CoCrFeNi	SLM	20–63	-	-	P = 150 W, v = 1600 mm s^−1^, t = 50 µm, h = 100 µm	FCC	-	520	~2	-	[73]
Al_0.5_CoCrFeNi	SLM	37–74	-	99.92	P = 320 W, v = 800 mm s^−1^, t = 60 µm, h = 50 µm	FCC + BCC	609	878	~0.175	270	[74]
AlCoCrFeNi	SLM	27–65	-	-	P = 120 W, t = 50–80 µm, h = 25 µm	BCC	-	-	-	541 ± 18	[75]
AlCoCrFeNi	SLM	-	-	-	P = 98 W, v = 2000 mm s^−1^, t = 52 µm	BCC + B2	-	-	-	-	[76]
AlCoCrFeNi	EBM	~70	-	-	Voltage = 60 kV, vacuum = 7 × 10^−1^ Pa of He, build plate temperature = 1173 K	FCC + BCC + B2	769 ± 12.7	1073.5 ± 21.3	1.2 ± 0.2	-	[77,78]
AlCoCrFeNiTi	SLM	22–65	-	-	P = 260 W, v = 1000 mm s^−1^, t = 50 µm, h = 70 µm	FCC + Oxides	1235	1550	10.69	-	[78]
Al_4_(CoCrFeNi)_94_Ti_2_	SLM	22–65	-	-	P = 400 W, v = 10 mm s^−1^, h = 460 µm	FCC	-	-	-	-	[79]
Al_0.5_Cr_0.8_CoFeNi_2.5_V_0.2_	SLM	15–75	-	~98.9	P = 250 W, v = 960 mm s^−1^, t = 40 µm, h = 80 µm	FCC	530 ± 15	1842 ± 35	40	263 ± 10	[80]
AlCrFeNiV	SLM	20–80	-	99.88	P = 140 W, v = 900 mm s^−1^, t = ~30 µm, h = 50 µm	FCC	651.36	~1057.47	30.3	-	[81]
FeCoCrNi	SLM	-	-	-	P = 200 W, v = 300 mm s^−1^, t = 20 µm	FCC	600	745	32	238	[65]
		-	-	-	P = 200 W, v = 300 mm s^−1^, t = 50 µm	FCC	402	480	8	205	[65]
		14–48	-	99.71 ± 0.25	P = 200 W, v = 740 mm s^−1^, t = 40 µm, h = 40 µm	FCC	581.9	707.9	20	218	[82]
		14–48	-	99.71 ± 0.25	Annealed at 1573 K for 2 h	FCC	221	633.2	45	138	[82]
FeCoNiCuAl	SLM	15–65	-	~96	P = 100–400 W, v = 400–1200 mm s^−1^, t = 40 µm, h = 80 µm	BCC matrix + Cu-rich B2	-	-	-	~858	[83]
Ni_6_Cr_4_WFe_9_Ti	SLM	15–53	-	-	P = 300 W, v = 2500 mm s^−1^, t = 100 µm, h = 80 µm	-	742	972	12.2	-	[84]
CoCrFeNiMn	EBM	45–106	-	-	Reference current = 2–14, scanning speed = 492–3446 mm/s), layer thickness = 50–70 µm, hatch depth = 50–70 µm	-	205 ± 3	497 ± 2	63 ± 1	157.1	[85]
CoCrFeNiW_0.2_	SLM	15–45	-	-	P = 175 W, v = 150 mm s^−1^, t = 30 µm, h = 100 µm	FCC + W	610 ± 15	814 ± 9	17 ± 1	314.1	[86]
CoCrFeMnNi	SLM	5–45	-	-	P = 400 W, v = 830 mm s^−1^, t = 30 µm, h = 90 µm	FCC	-	601	35	-	[87]
	SLM	-	-	-	P = 400 W, v = 830 mm s^−1^, t = 30 µm, h = 90 µm then HIP temp 1150 °C, time 3 h, and pressure 150 MPa	FCC	-	649	18	-	[87]
	SLM	-	-	99.2	P = 240 W, v = 2000 mm s^−1^, t = 40 µm, h = 50 µm	FCC	510 ± 10	609 ± 10	34 ± 3	-	[88]
	SLM	5–45	-	99.2	P = 240 W, v = 2000 mm s^−1^, t = 40 µm, h = 50 µm then HT at 900 °C for 1 h in Ar atmosphere	FCC	381 ± 8	619 ± 5	47 ± 2	-	[88]
CrFeNiMn	SLM	20–45	-	98.0% ± 0.1	P = 70 W, v = 200 mm s^−1^, t = 25 µm, h = 90 µm	FCC	-	-	-	248 ± 8	[89]
NbMoTaW	SLM	-	-	-	P = 400 W, v = 250 mm s^−1^, t = 100 µm, h = 100 µm	-	-	-	-	826	[90]
Ni_6_Cr_4_WFe_9_T	SLM	15–53	-	-	P = 300 W, v = 2500 mm s^−1^, t = 100 µm, h = 80 µm	-	742	972	12.2	-	[84]

P = power (W), v = scan speed (mm s^−1^), t = layer thickness (µm), and h = hatching space (µm).

**Table 5:** The mechanical properties (compressive strength and hardness) of various Ni-containing high entropy alloys.

HEAs System	AM Techniques	Powder Size (μm)	Density	Relative Density	Printing Parameters	Phase Compositions	Yield Compressive Stength (Mpa)	Compressive Fracture Stength (Mpa)	Elongation (%)	Hardness (HV)	Ref.
AlCoCrFeNi	EBM	~70	-	-	Voltage = 60 kV, print orientation 0 degrees	FCC + BCC	1015.0 ± 52.5	1668.3 ± 71.5	26.4 ± 6.7	-	[91]
AlCoCrFeNi	EBM	~70	-	-	Voltage = 60 kV, print orientation 90 degrees	FCC + BCC	944.0 ± 55.4	1447.0 ± 135.8	14.5 ± 5.3	-	[91]
Al_2.1_Co_0.3_Cr_0.5_FeNi_2.1_	WAAM	-	-	-	Wire feed speed 8 m/min, voltage 17 V, travel speed 0.3 m/min, substrate heating temperature ≈ 250 °C	A2 + B2	550	1899	18.5	463	[92]
AlCoCrFeNi	BJ	~32	-	-	50 μm layer thickness, recoating speed of 1 mm/s, binder saturation 60%, heating power of 50%, and a drying time of 25 s, sintering at 1320 °C for 4 h, annealing at 1000 °C	FCC + BCC + B2	1203 ± 22	1996 ± 45	32.25 ± 2.5	418.1	[93]
AlCoCrFeNi	BJ	~32	-	-	50 μm layer thickness, recoating speed of 1 mm/s, binder saturation 60%, heating power of 50%, and a drying time of 25 s, sintering at 1320 °C for 4 h, annealing at 1200 °C	FCC + BCC + B2	1461 ± 23	2272 ± 48	31.46 ± 2.1	499.6	[93]
AlCrCuFeNi	SLM	11–58	-	-	P = 300 W, v = 600 mm s^−1^, t = 40 µm, h = 80 µm	BCC	-	2052.8 ± 123.6	6.8 ± 1.3	-	[94]
AlCrCuFeNiW_1_	LMD	90–150	-	-	P = 1600 W, v = 20 mm s^−1^, t = 40 µm	FCC + BCC	998.4	1274.6	24.3	240 ± 10	[95]
AlCrCuFeNiW_3_	LMD	90–150	-	-	P = 1600 W, v = 20 mm s^−1^, t = 40 µm	FCC + BCC	1005.9	1287.9	23.4	230 ± 10	[95]
CoCrFeMnNi	SLM	18–38	-	-	P = 90 W, v = 600 mm s^−1^, t =25 µm, h = 80 µm in scanning direction	FCC	778.4	-	-	-	[70]
CoCrFeMnNi	SLM	18–38	-	-	P = 90 W, v = 600 mm s^−1^, t = 25 µm, h = 80 µm in transverse direction	FCC	766.4	-	-	-	[70]
CoCrFeMnNi	SLM	18–38	-	-	P = 90 W, v = 600 mm s^−1^, t = 25 µm, h = 80 µm in building direction	FCC	703.5	-	-	-	[70]

P = power (W), v = scan speed (mm s^−1^), t = layer thickness (µm), and h = hatching space (µm).

The majority of AM-processed HEAs demonstrate adherence to the Hall–Petch relationship, showing an inverse correlation between yield strength and average grain size. Achieving remarkable strength alongside required ductility has been realized by avoiding the creation of brittle intermetallic phases via compositional tuning and/or thermomechanical processing. For example, the as-deposited CoCrFeNi HEA via LMD reached an elongation of 92.5% alongside strength surpassing its cast analogue [96]. Alloying HEAs with interstitial elements (C, N, Si) through AM technique power flexibility synergizes strength and ductility. The improvements originate from varied mechanisms including nano-scale solute clustering, dislocation slip, deformation twinning, and strain-prompted phase transformations. Certain metastable HEAs like Fe_60_Co_15_Ni_15_Cr_10_ showcase high tensile ductility after first necking owing to the steady spread of strain-generated martensitic transformation domains. For example, an as-printed sample of this alloy composition exhibited 69.5% elongation at acceptable strength levels [97]. Eutectic HEAs processed via AM form alternating nano-lamellar microconstituents spacing within colonies, conferring simultaneous high yield strength (~1000 MPa) and reasonable tensile ductility (~20%) [98,99,100,101]. Their impressive strength–ductility balance highlights their candidacy for AM-related structural applications. In summary, AM provisionally enables extensive microstructure and composition tailoring avenues to develop HEAs with excellent strength-ductility synergies for load-bearing components. The careful selection of AM method and tuning fabrication parameters alongside judicious alloying additions can elicit optimized tensile properties.

### 4.3. Compressive Properties

Compressive strength analysis in HEAs additive manufacturing is essential for determining material appropriateness for parts that could be compressed, including those in the automobile or aerospace sectors. The optimization of HEA composition and printing settings is guided by compressive strength data, guaranteeing that constructions possess the requisite strength for their designated purpose. It is essential for assessing structural integrity, performing failure analysis, and arriving at well-informed design choices. Compressive strength is a statistic used in quality control operations that helps with material certification and standardization in additive manufacturing. Furthermore, these data help with predictive modeling, which enables engineers to estimate how HEA constructions will behave under different compressive stress scenarios. The compressive strength, hardness, phase composition, printing parameters and powder size to produce various Ni-containing alloys are listed in Table 5.

**Figure 4:**
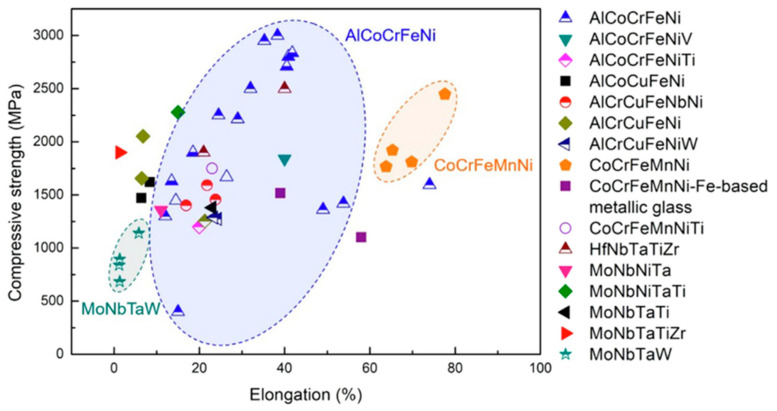
The fracture elongation and compressive strength of several AMed high-energy alloys. The HEAs manufactured by SLM, LMD, EBM, and WAAM are represented by the solid, top half-solid, open, and bottom half-solid symbols, respectively (only considering the as-fabricated state) [60]. Adapted with permission from ref. [60]. A 2022 Creative Commons Attribution (CC BY) license.

Figure 4 shows a comparison between the different types of Ni-based HEAs obtained from various studies. Fujieda et al. showed electron-beam-melted (EBM) AlCoCrFeNi had much higher deformability (1668 MPa compressive strength) compared with cast AlCoCrFeNi, ascribed to the finer grains and enhanced ductility of the FCC-structured phase from EBM [91]. Meanwhile, increasing Al content in laser-metal-deposited (LMD) Al_x_CoCrFeNi increased compressive strength but reduced ductility [54,102]. Al_0.3_CoCrFeNi sustained high work hardening rates exceeding 1300 MPa compressive strength with a strain of 1 due to deformation twinning, while BCC Al_0.85_CoCrFeNi showed higher power (2250 MPa) but lower elongation (24.5%) due to second phase particles [103]. Significant tension-compression property differences were reported in LMD Al_0.3_CoCrFeNi as well, related to the absence of deformation twins in tension [103]. Anisotropy in properties was also found in selective laser melted (SLM) CoCrFeMnNi due to differences in dislocation and twinning behavior along different directions [70]. A yield strength prediction model involving grain size, oxides, and orientation-dependent Taylor factors matched these experiments. Similar anisotropy occurred in LMD AlCoCrFeNi_2.1_ and SLM AlCrCuFeNi during compression testing [94,100]. For refractory HEAs, LMD MoNbTaW (W = 0) exhibited an 874 MPa yield strength and a 1140 MPa compressive strength. Meanwhile, laminated LMD AlCoCrFeNiTi/CoCrFeMnNi showed yield strengths of up to 990 MPa and no fractures at strains as large as 80%, significantly outperforming monolithic HEAs. This demonstrates AM’s capability to create HEAs with enhanced property combinations [68]. It is important to note that AM HEAs can attain high compressive strengths exceeding 2 GPa but anisotropy and a lack of strain causing failure are persistent challenges. Alloy and process optimization to promote twinning and fine, uniform microstructures is critical, as is the exploration of laminated architectures that can achieve synergistic strengthening across interfaces.

Lopes and Oliveira studied the Optimization of Process Parameters in Laser Powder Bed Fusion of SS 316L Parts Using Artificial Neural Networks [104]. To find the optimal process parameters for user-required part properties in laser powder bed fusion, an optimization model based on experimental data was developed using artificial neural networks. An exclusive neural network was developed to optimize the laser power, scan speed, and hatch spacing with the desired relative density, surface roughness, dimensional error, and microhardness. Their predicted values were compared with the experimental values in the literature and indicated a close match. They also demonstrated the ability of an exclusive neural network in modeling the process parameter–property relationships for multiple properties. The developed model can find the optimal processing parameters that satisfy the user requirement for customized part properties. Thus, it reduces the preprocessing time and cost significantly. The following conclusions are drawn based on the current study. Finding the optimal parameters for the laser powder bed fusion process requires an understanding of the combined effect of the process parameters on the part properties. ANN is a powerful tool in modeling the combined relationship and obtaining the optimal process parameters in the given range of data.

### 4.4. Porosity

One of the leading causes of the uneven porosities inside the AM components is inadequate melting due to incomplete fusion. Still, there are other factors as well that raise serious concerns. When there is not enough fusion, the melt pool does not go far enough into the preceding layer to facilitate the formation of metallurgical bonds with the subsequent layers [62]. Underneath the present layer, un-melted and unconsolidated powder remains trapped. The remaining powdered areas then appear as pores in the finished product. Optimizing processing parameters to obtain sufficient energy density and melt pool penetration for complete consolidation without excess is necessary to reduce the lack of fusion [105].

Islam et al. [106] studied six metallic powders characterized in terms of PSD using a range of techniques and concluded that spherical powder produced more acceptable results over the range of equipment used. Larger differences arose as the morphology deviated away from spherical. Sofia et al. [107] reported that at a fixed scan velocity and power of the laser, the melting of the powders was significantly affected by their size. Yuan et al. [108] studied SLM-manufactured GH3230 nickel-based superalloys and explored the impacts of various post-processing on the microstructural advancement and extensive mechanical properties of the GH3230 superalloy. They reported that after HIP treatment, the liquid pool structure vanished, and the microstructure principally comprised columnar grains.

**Figure 5:**
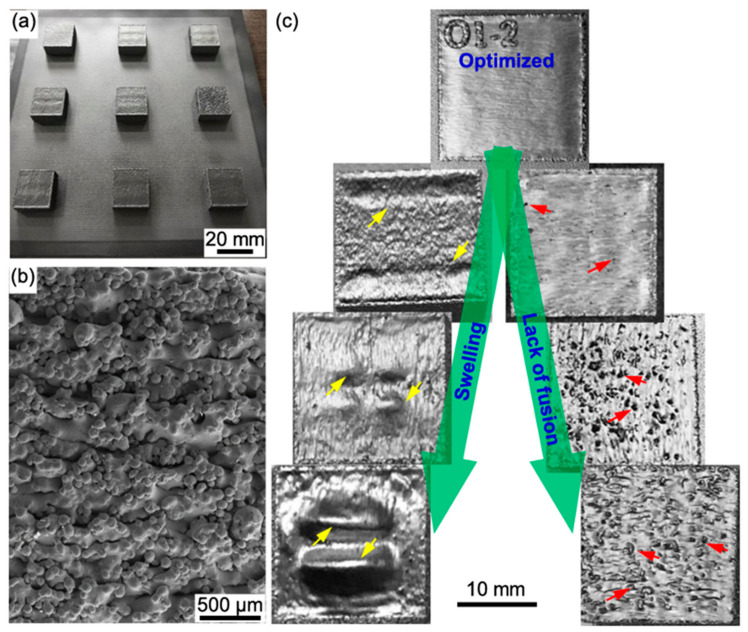
(**a**) shows a part made using EBM, (**b**) an SEM image showcasing the rough surface texture from a side view, and (**c**) a representation of the top surface of cuboid samples exhibiting swelling and lack of fusion. Swelling is denoted by yellow arrows, while areas of incomplete fusion are highlighted by red arrows [85]. Adapted with permission from ref. [85]. A 2019 Creative Commons Attribution (CC BY) license.

The size and penetration of the melt pool were influenced by several parameters, including the preheat temperature, scanning speed, focus offset, and beam current. Figure 5 shows images of the as-built EBM CoCrFeNiMn HEA parts. Figure 5a shows the whole part with surrounding powders adhered to the sides, indicating non-optimal contour processing parameters and roughness. Figure 5b shows the rough side surface finish under SEM. Figure 5c shows the characters’ cuboid sample top surfaces exhibiting varying swelling and lack of fusion defects. Swelling occurs when process parameters cause huge melt pools and high temperatures. An absence of fusion defects occurs when there is insufficient energy density to fully melt and fuse each layer.

**Figure 6:**
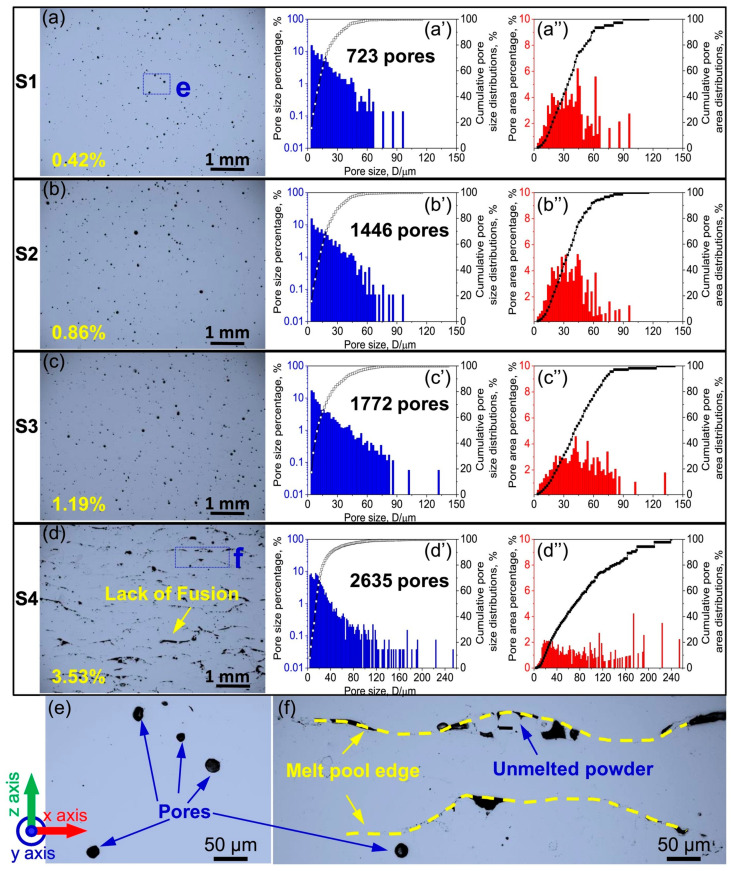
(**a**–**f**) OM images of EBM-built specimens with corresponding (**a′**–**d′**) pore size and (**a″**–**d″**) pore area distributions. (**a**,**a′**,**a″**) stand for specimens 1 (S1), 2 (S2), 3, and 4, respectively. (**c**,**c′**,**c″**) stand for specimens 3 (S3) and 4 (S4), respectively. Enlarged pictures from (**a**,**d**) in (**e**,**f**) correspondingly reveal the presence of gas pores and the absence of fusion faults [85]. Adapted with permission from ref. [85]. A 2019 Creative Commons Attribution (CC BY) license.

Porosity is a challenge in additively manufactured parts, which is very important to take care of while printing. Figure 6 illustrates the significant impact of feedstock powder quality on the final porosity levels in additively manufactured CoCrFeNiMn HEA parts built by EBM [85]. Optical microscopy reveals that most pores in the EBM samples match the argon gas pores captured within the gas-atomized powder particles utilized as the powder bed feedstock. More pores in the powder means more pores in the printed part. Furthermore, a lack of fusion defects manifests as larger pores during EBM processing if the energy density is insufficient to fully consolidate each layer, sharply escalating porosity. Wang et al. [85] demonstrated that while the gas-atomized alloy powder itself exhibited suitable morphology and flowability for EBM, the optimization of the EBM process parameters was essential to minimize both types of pores and achieve 99%+ relative density. This extensive parameter tuning would be considerably significantly reduced with high quality, pore-free powder feedstock. In summary, Figure 6 signifies that powder quality fundamentally constrains the final part quality, and eliminating internal gas pores in the atomization process is critical to enable the reliable, efficient printing of high-performance additively manufactured metal alloys [85].

## 5. Effect of VEC on Ni-Based HEAs

**Figure 7:**
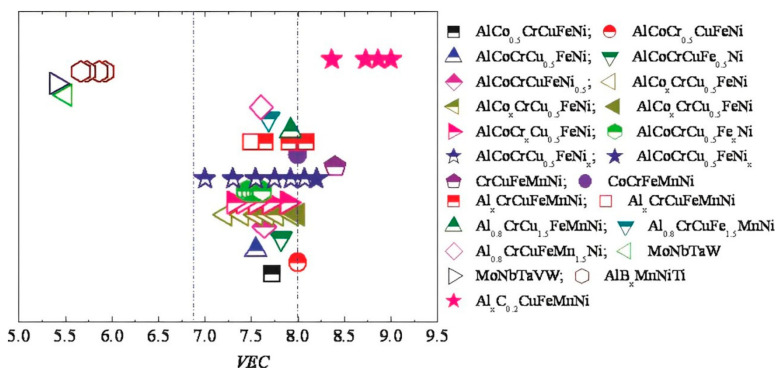
Co-relation of VEC with the crystal structure (BCC or FCC) [109]. Reprinted with permission from ref. [109]. © 2016 AIP Publishing.

The structural properties of HEAs are influenced by their microstructure, which is significantly determined by valence electron concentrations (VEC) of the HEA composition [109]. The VEC is determined from the statistical average of VECs of constituting elements of the HEA. Guo et al. [109] explored the relationship between VEC and crystal structure in various HEAs as depicted in Figure 7. Their findings suggest that alloys with a VEC greater than 8 tend to stabilize in the FCC phase, those with a VEC between 6.87 and 8 exhibit a mixture of FCC and BCC phases, and those with a VEC less than 6.87 favor the BCC phase. However, this rule has no consideration for HCP-structured alloys and physically it is impossible for all outer shell electrons to take part in bonding process; moreover, the VEC rule provides a crude solution to predict crystal structure of HEAs.

**Figure 8:**
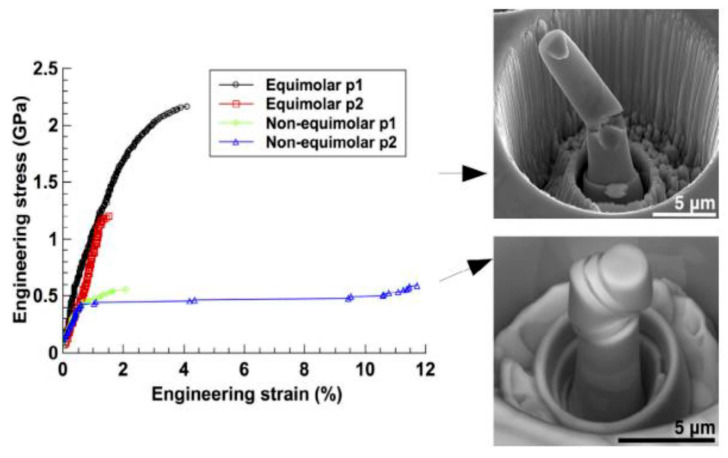
Variation of tensile properties due to change in crystal structure i.e., BCC to FCC phase [110]. Reprinted with permission from ref. [110]. © 2017 Elsevier.

Vida et al. [110] reported two different HEAs, one Ni_35_Fe_30_Cr_20_Mo_10_W_5_ which is equiatomic, and Ni_35_Fe_30_Cr_20_Mo_10_W_5_, which is non-equiatomic. The VEC values for these alloys were calculated to be 7.2 and 8, respectively. With a VEC value around 8, both alloys exhibited a mixture of phases, but their structures could shift between BCC and FCC, affecting their properties, as illustrated in Figure 8. Thus, when the VEC approaches 8, the FCC phase becomes more dominant.

**Figure 9:**
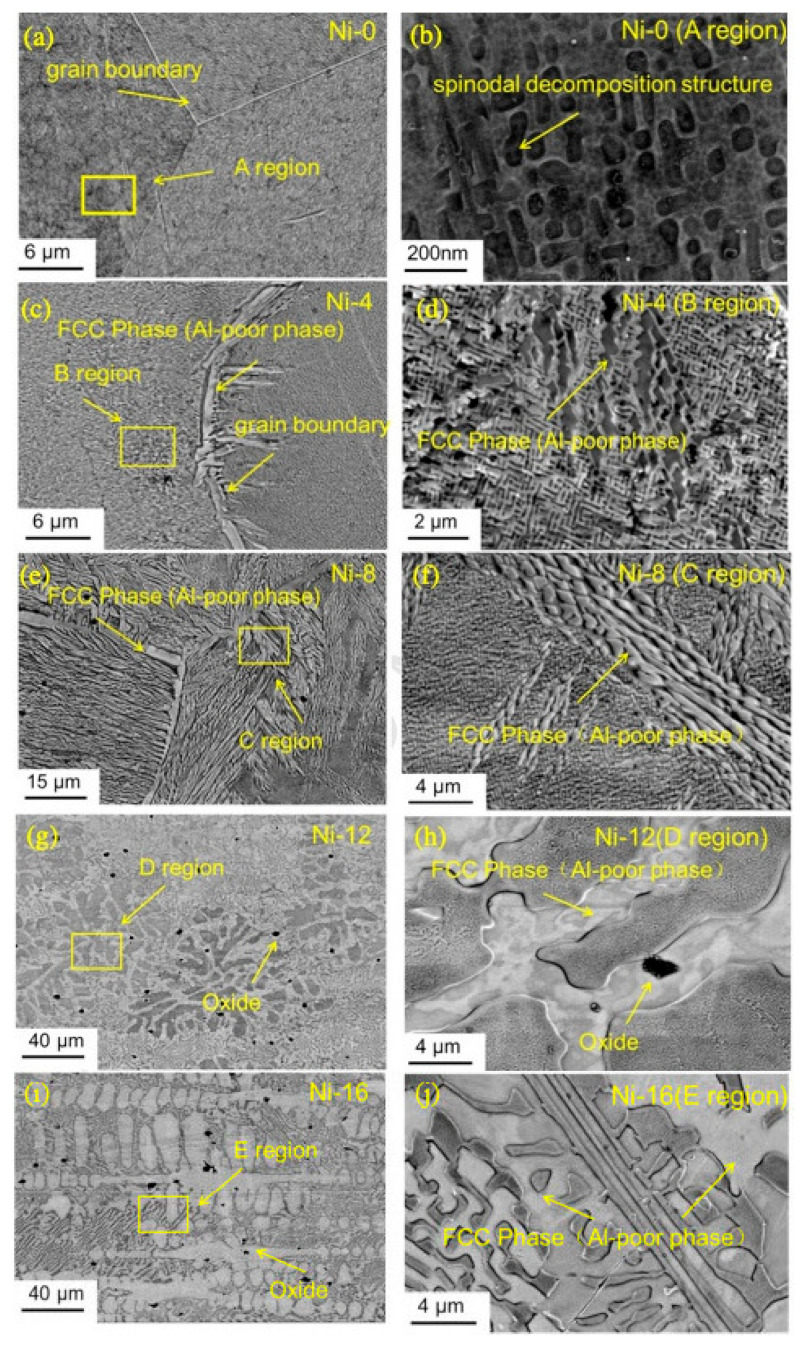
SEM of as-cast (AlCoCrFeNi)_100−x_Ni_x,_ where x is (1/4, 0, 4, 8, 12, and 16) at corresponding low and high magnification [111]. Reprinted with permission from ref. [111]. © 2018 Elsevier.

**Figure 10:**
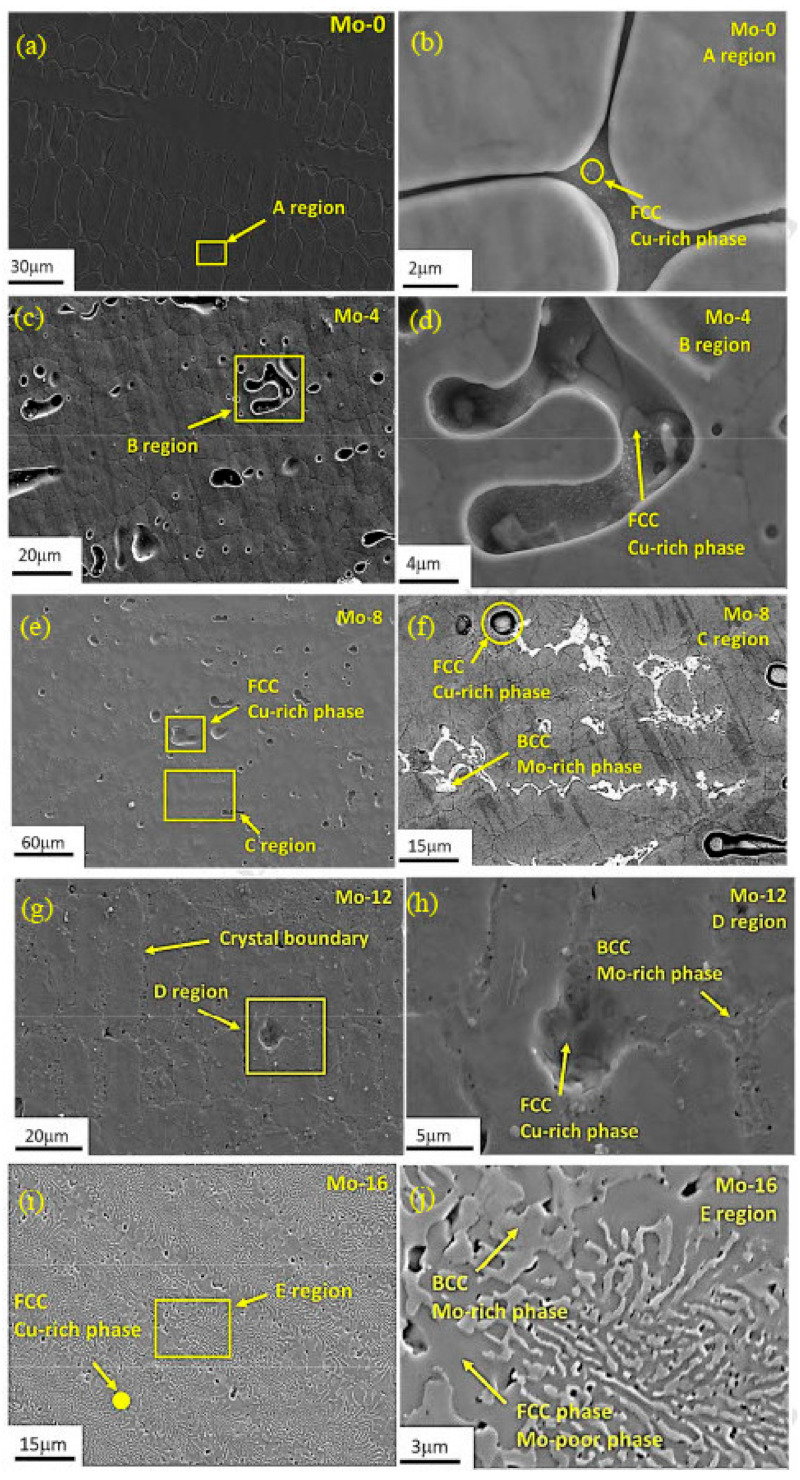
Scanning Electron Micrograph (SEM) of as-cast (CoCrCuFeNi)_100−X_Mo_x_, where x is (1/4, 0, 4, 8, 12, and 16) at corresponding low and high magnification [111]. Reprinted with permission from ref. [111]. © 2018 Elsevier.

Synthesizing HEAs requires balancing between strength and ductility. Higher VEC tend to promote FCC structures, which enhance ductility but often at the cost of strength. To optimize the power and flexibility, Chen et al. [111] aimed to enhance strength and ductility by alloying Ni and Mo within two different types of HEAs, namely (AlCoCrFeNi)_100−x_Ni_x_ and (CoCrCuFeNi)_100−x_Mo_x_, by utilizing VEC to tailor the phase composition. It is important to note that AlCorFeNi exhibits a BCC structure and CoCrCuFeNi exhibits an FCC structure. It was seen that when increasing the Ni percentage in the alloy (AlCoCrFeNi)_100-x_Ni_x_, the VEC shifts towards the FCC structure, as illustrated in Figure 9. When the Ni content varies from 0% to 16%, the compressive fracture strain improves from 25% to 40%. Likewise, as the Mo percentage increases in the (CoCrCuFeNi)_100−x_Mo_x_, the VEC shifts towards the BCC structure, as illustrated in Figure 10. The compressive yield strength showed a consistent increase from 260 MPa to 928 MPa as the Mo content varied from 0% to 16%.

Figure 9 shows SEM micrographs of as-cast HEAs with varying Ni percentages, demonstrated in the left column. The regions identified in the left column are magnified and displayed in greater detail in the corresponding areas on the right column. Figure 9a has confirmed that the AlCoCrFeNi HEA exhibits a uniform, single phase structure. The enlarged SEM image in Figure 9b (for labeled area A in Figure 9a) resembles honeycomb-like structures with uniformly distributed spherical particles. The reported microstructures matched the microstructures of AlCoCrFeNi HEA synthesized by Bridgman solidification [112]. Additionally, they resembled the as-cast Fe-Ni-Mn-Al alloy microstructure [113]. Figure 9c shows the microstructures for the (AlCoCrFeNi)_96_Ni_4_ HEA. It reveals the existence of FCC phases with a deficiency of aluminum, noticeable at the boundaries of the grains. These phases were also observed within the grains themselves, as depicted in Figure 9d. Notably, their small volume fraction made them undetectable using XRD techniques. The microstructures depicted in Figure 9e,f reveal the characteristics of the (AlCoCrFeNi)_92_Ni_8_ HEA. In the (AlCoCrFeNi)_96_Ni_4_ HEA, an aluminum-deficient composition, FCC phases of notable size were identified, surpassing those observed in the (AlCoCrFeNi)_96_Ni_4_ HEA. Figure 9g–j illustrate the microstructures of the (AlCoCrFeNi)_100−_*_x_*Ni*_x_* HEA specifically for x values of 12 and 16. The SEM micrographs indicated a gradual growth of precipitated FCC phases as the percentage of Ni increased, highlighting an Al-deficient composition. This observation suggests a positive influence of Ni on the transformation from BCC to FCC, given its role as an FCC stabilizer. Within the as-cast AlxCoCrFeNi alloy, using varying concentrations of Al as the alloying element in CoCrFeNi, it was found that Al significantly enhances the mechanical properties by stabilizing the BCC structure of the Al-rich alloy as compared with Al-deficient alloys [114,115,116]. In principle, the mechanical properties are dependent on their microstructures and this alloy also exhibits various crystal structures and microstructures depending on the aluminum content, ranging from FCC structures at low Al content levels to BCC phases with spinodal decomposition at higher Al content levels. The microstructure evolves from columnar cellular and dendritic structures to equiaxed nondendritic and dendritic grains, with the Widmanstätten side plates and complete spinodal structures forming at specific Al content levels [114].

The black regions in Figure 9g–i were detected as O with EDS. The origin of oxygen in the system, whether from the initial raw material or during subsequent processing stages, remained unclear. The minuscule sizes of oxide particles had no discernible impact on the structural properties of HEAs. The selection of Mo, with a lower VEC compared with the average VEC value for the CoCrCuFeNi alloy, was selected as an element for incorporation into the CoCrCuFeNi matrix to prove the feasibility of the alloy design. Figure 10 shows the micrographs of the as-cast alloy with varying Mo percentages.

Figure 10a shows single phase CoCrCuFeNi HEA. As seen in the high magnification micrograph in Figure 10b (focused on area A in Figure 10a), Cu-rich phases were discovered along the grain boundaries, as depicted in Figure 10b–d, which display micrographs at varying magnifications for the (CoCrCuFeNi)_96_Mo_4_ HEA. Within the grains, Cu-rich phases were observed, highlighting the distribution within the material. Figure 10e,f showcase magnified microstructures from the (CoCrCuFeNi)_92_Mo_8_ HEA. In the microstructures, BCC phases containing Mo are evident, with their volume fraction appearing to rise alongside the Mo percentage, as depicted in (Figure 10g–j). This suggests a beneficial impact of Mo on the transition from FCC to BCC.

## 6. Pressure-Induced FCC to HCP Phase Transition in Ni-Based HEAs

**Figure 11:**
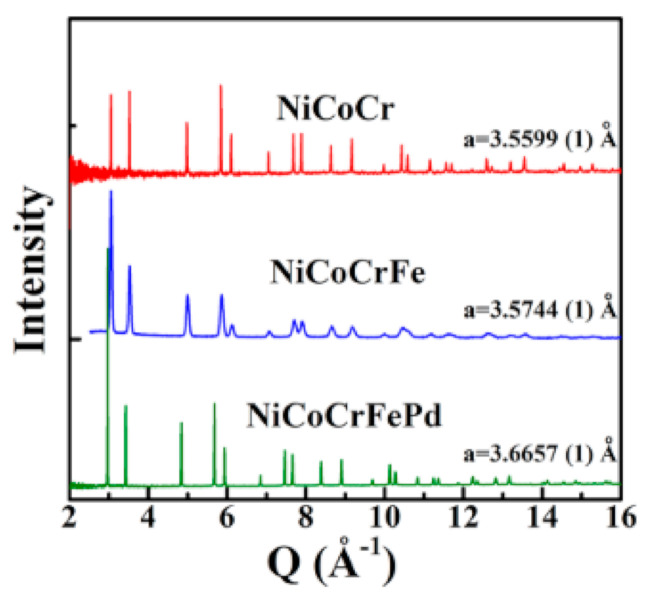
Neutron diffraction profiles depicting single phase FCC solid solution for alloys “NiCoCr”, “NiCoCrFe”, and “NiCoCrFePd” [117]. Reprinted with permission from ref. [117]. © 2017 AIP Publishing.

Ni-based high entropy alloys with an FCC structure can undergo a phase transformation to an HCP structure under extremely high pressures. This pressure-induced FCC to HCP phase transition has significant implications for the mechanical properties and potential applications of these multi-principal element alloys. Zhang et al. [117], investigating the transition of FCC to HCP phase via pressure-induced strains, proposed that this has to be a martensitic transformation. These mechanisms require less activation energy. In this study, three HEAs were arc melted and drop cast, namely, NiCoCr, NiCoCrFe, and NiCoCrFePd, from individual metal powders of high purity (>99.9%). All alloys were found to crystalize into a face-centered cubic structure, as presented in Figure 11.

**Figure 12:**
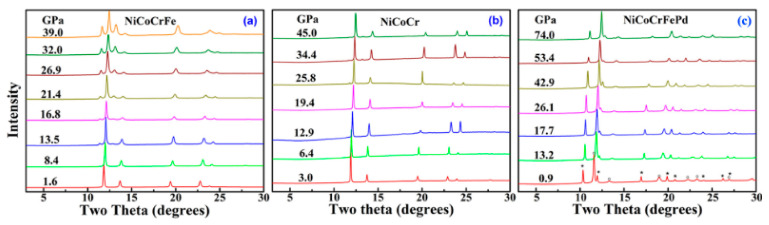
(**a**–**c**) shows XRD patterns for NiCoCrFe, NiCoCr, and NiCoCrFePd alloys under high pressure, illustrating the transformation from FCC to HCP structure at pressures of 13.5, 45, and 74 GPa, respectively [117]. The diffraction peaks marked with * are from Au pressure marker. Reprinted with permission from ref. [117]. © 2017 AIP Publishing.

Synchrotron XRD studies revealed that these HEAs undergo a lethargic FCC to HCP transition at high pressure. Figure 12a–c shows the XRD profile of “NiCoCrFe”, “NiCoCr”, and “NiCoCrFePd” alloys, respectively. The NiCoCrFe alloy exhibits an FCC–HCP transition at around 13.5 GPa. Similarly, ternary alloy “NiCoCr” undergoes a transformation from FCC to HCP at 45 GPa while quinary alloy “NiCoCrFePd” shows transformation from FCC to HCP at a much higher pressure of 74 GPa. The diffraction peaks marked with * are from Au pressure marker as shown in Figure 12c. The sluggish nature of this polymorphic transition and the varying transition pressures across different alloy compositions highlight the importance of elemental chemistry while down selecting elements for HEAs.

HCP and FCC structures show densely packed arrangements of atoms, contrasting each other only in stacking order [118]. Studies found that alloys underwent an HCP shift because of defects caused by pressure, resulting in altered stacking fault from ABCABC… to ABAB… This aligns with the lower stacking fault energy observed in HEAs [119,120].

## 7. Precipitation-Strengthened Ni-Based HEAs

HEAs are known to exhibit remarkable strength while maintaining superior ductility. Precipitation strengthening mechanisms hold promising potential in this domain, offering avenues for tailoring the required properties in these alloys. Tsao et al. [121] developed a cost-effective method to produce Ni-based HEAs exhibiting exceptional high-temperature performance on par with traditional Ni-based superalloys. Their approach aimed to create advanced materials that could withstand extreme thermal conditions while maintaining affordability and practicality. For high temperature applications, the alloy must exhibit exceptional structural integrity and oxidation resistance at elevated temperatures [122,123]. To stabilize the surface in Ni-based HEAs, L1_2_ Ƴ’ precipitate was procured, which is attributed to the coherency of Ƴ’ with the matrix [124]. Moreover, intermetallics/ordered phases like σ, µ, η, etc., tend to make HEAs brittle due to principate-induced higher interfacial energy with the matrix [125]. Tsao et al. [121] synthesized two HEAs, as tabulated in Table 6**.**

**Figure 13:**
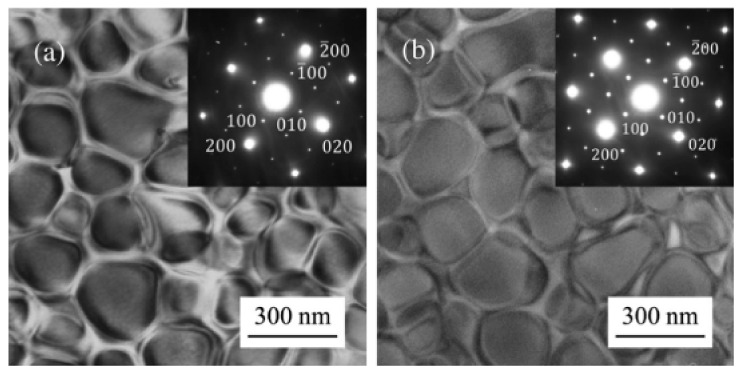
(**a**,**b**) Displays TEM bright field images with insets showing diffraction spots at the [001] zone axis for the alloys HESA-1 and HESA-2, respectively [121]. Reprinted with permission from ref. [121]. © 2017 John Wiley and Sons.

The microstructure in Figure 13 shows that, after solution and aging treatment, the Ƴ’ uniformly dispersed precipitates within the FCC Ƴ matrix. Further, the inset diffraction pattern confirms the presence of an L12 ordered γ’ phase, with no other degrees detected, which is comparable to conventional Ni-based superalloys [124]. Both HE-Superalloys exhibit Ƴ’ precipitate with spherical morphology.

Table 7 shows the composition of Ƴ/Ƴ’ of HESA and some common Ni-based superalloys [121]. Ƴ and Ƴ’ are Ti-rich solid solutions, with Ƴ’ having a higher Ti content. Additionally, Fe promotes partitioning between Ƴ and Ƴ’, causing lattice misfit, which enhances strengthening by increasing the anti-phase boundary energy. Moreover, the incorporation of less refractory elements and high-temperature stability might exceed that of commercial superalloys.

## 8. Substitute for TRIP and TWIP Steels via Ni-Based HEAs

**Figure 14:**
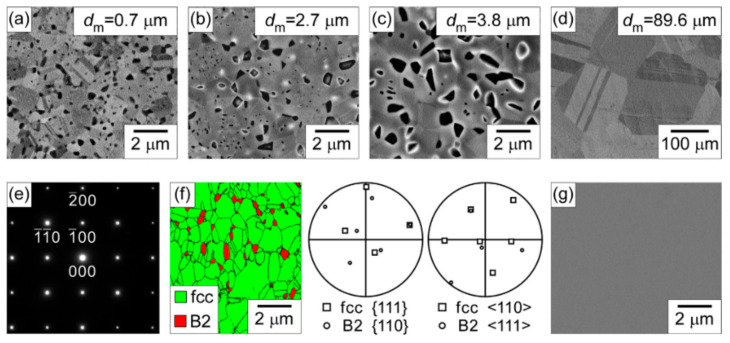
(**a**–**d**) show SEM-BSE micrographs of samples that underwent recrystallization for 1 h at temperatures of 1073 K, 1173 K, 1273 K, and 1373 K, respectively. These micrographs provide insights into the microstructural evolution during the recrystallization process at different temperatures; (**e**) displays the SAED pattern obtained from a B2 precipitate phase present in the material. SAED is a technique used to analyze the crystal structure and orientation of localized regions within a sample; (**f**) presents an EBSD image illustrating the distribution of face-centered cubic (FCC) and B2 phases within the material. Accompanying the EBSD image are pole figures, which represent the crystallographic orientations of the respective phases; (**g**) shows a BSE micrograph of a single crystal sample that was annealed at 1273 K for 100 h. This micrograph provides information about the microstructural changes induced by the prolonged heat treatment [129]. Reprinted with permission from ref. [129]. © 2017 Elsevier.

Ni-based HEAs have emerged as promising candidates to substitute transformation-induced plasticity (TRIP) and twinning-induced plasticity (TWIP) steels, offering superior mechanical properties and corrosion resistance [126,127]. These multi-principal element alloys leverage compositional complexity to achieve exceptional strength-ductility synergy through tailored stacking fault energies and deformation mechanisms. In particular, Al_x_CoCrFeNi alloys typically exhibit either FCC or BCC structures, contingent to the values of x [114,128]. Notably, Yasuda and colleagues [129] designed the Al_0.3_CoCrFeNi alloy, synthesized using a plasma arc furnace, and revealed the presence of a NiAl phase with a B2 structure precipitated along the grain boundaries. During cold rolling, these fine precipitates were found to pin the grain boundaries, thereby inhibiting grain growth. Figure 14 presents the SEM backscattered electron micrographs and their pole figures.

Figure 14a–c illustrate that precipitates primarily form along grain boundaries at temperatures up to 1273 K. However, at 1373 K, these precipitates tend to dissolve. Figure 14e presents the SAED pattern of the residue, and on subsequent EBSD analysis in Figure 14f, identifies the B2 structure dispersed within the FCC matrix. A noteworthy observation was made during the annealing of a single crystal Al0.3CoCrFeNi alloy: no B2 precipitates appeared even after 100 h at 1273 K. This indicates that the B2 structure is inclined to form at grain boundaries.

**Figure 15:**
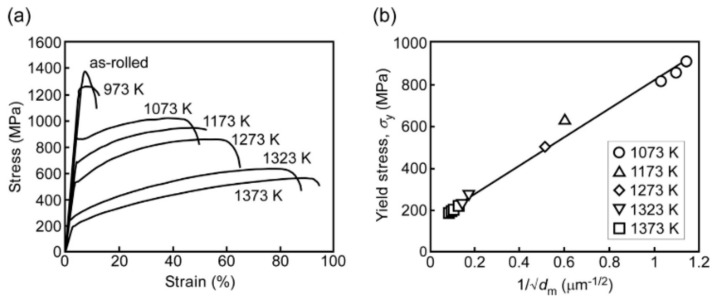
Presents (**a**) the stress−strain curve and; (**b**) the Hall−Petch relation for Al_0.3_CoCrFeNi HEA at different annealing temperatures [129]. Reprinted with permission from ref. [129]. © 2017 Elsevier.

The mechanical properties of the alloy were analyzed and are presented in Figure 15a,b. As illustrated in Figure 15a the alloy annealed at temperature at or below 1273 K displays a high yield stress exceeding 500 MPa. Additionally, an optimal combination of ultimate yield strength (over 1 GPa) and ductility (over 40 percent) is observed at an annealing temperature of 1073 K, which is equivalent to that of transformation-induced plasticity (TRIP) and twinning-induced plasticity (TWIP) [129].

In Figure 15b, yield stress is plotted against $\frac{1}{\sqrt{d_{m}}}$ for the recrystallized alloys annealed between 1073 K and 1373 K. Despite the absence of precipitates at temperatures beyond 1273 K, a linear trend is still observed. This indicates that grain boundary hardening remains the primary factor contributing to the enhanced strength for recrystallized Al_0.3_CoCrFeNi alloy.

## 9. Thermal Shock and Oxidation Resistance on Ni-Containing HEA

The mechanical characteristics of alloys made from one or multiple solid solution phases are distinctive. Studies show that BCC-structured HEA systems have excellent hardness and strength under compression [17,130,131], whereas FCC-based HEAs exhibit excellent plasticity.

**Figure 16:**
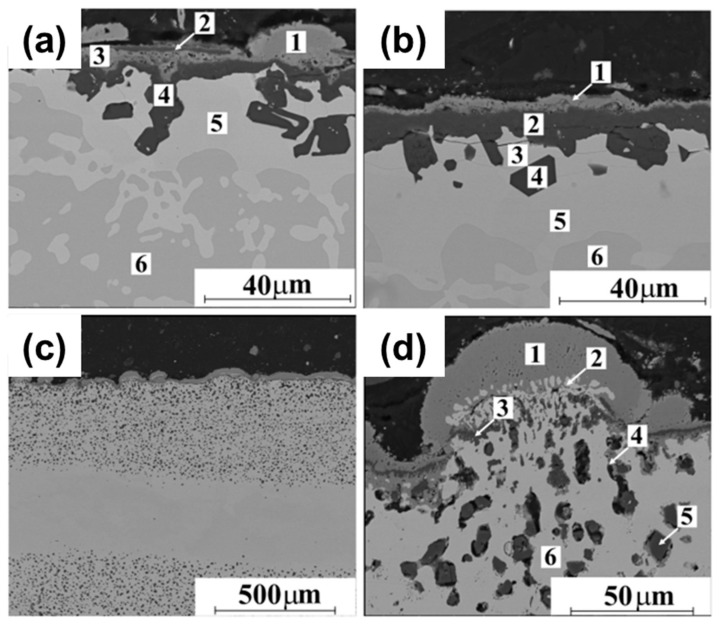
SEM cross-section images of (**a**) Al_25_Co_25_Cr_25_Ni_25_, (**b**) Al_20_Co_25_Cr_25_Ni_25_Si_5_, and (**c**,**d**) A_l9.1_Co_18.2_Cr_18.2_Fe_18.2_Ni_36.3_ prior to oxidation under thermal shock state at 1273 K in air [132]. Reprinted with permission from ref. [132]. © 2022 Elsevier.

By arc melting in a tungsten electrode-based melting chamber with inert atmosphere resting on a water-cooled copper plate, elemental powders of 99.99 wt% purity were combined in molar ratios chosen for enabling the production of alloys with the elemental percentages stated in Table 8 [132]. High purity Ti powder is generally employed as a getter. Resultant pallets are then remelted five times to acquire appropriate homogenization. The samples were then cut into discs with a diameter of about 150 mm and 1.5 mm thick, using a precision saw. Following these preparations, the samples underwent cyclic oxidation in a normal environment at 1273 K. Each phase of the thermal shock cycles, involving heating and cooling, lasted for a duration of 2 h. The heating and chilling processes each took about a minute. Throughout the oxidation process, more than 500 cycles (or more than 1000 h) were completed [132]. Figure 16a,b, respectively, exhibit SEM micrographs of the cross-sections for Al_25_Co_25_Cr_25_Ni_25_ and Al_20_Co_25_Cr_25_Ni_25_Si_5_ after cyclic oxidation at 1273 K, and Figure 16c,d show images of A_l9.1_Co_18.2_Cr_18.2_Fe_18.2_Ni_36.3_ at two different magnifications. Table 9 contains the EDS map findings from the zones shown in Figure 16a,b,d. These show chemical compositions discovered using EDS point analysis on Al_25_Co_25_Cr_25_Ni_25_, Al_20_Co_25_Cr_25_Ni_25_Si_5_, and A_l9.1_Co_18.2_Cr_18.2_Fe_18.2_Ni_36.3_ cross-sections following oxidation under thermal shock state at 1273 K in atmospheric environment.

According to Table 8, the thin intermediate layer of the scale developed on Al_25_Co_25_Cr_25_Ni_25_ predominantly comprises aluminum and oxygen, with trace amounts of the other constituent elements. In contrast, the outer layer of the scale is primarily composed of chromium and oxygen. Underneath this layer, where considerable levels of Al are also found, the oxide scale has significantly higher concentrations of Cr, Co, and Ni [132]. There is an Al-rich layer at the scale/substrate contact (dark phase zone). This dark zone is in connection with a bright degree consisting primarily of Ni, Co, and Cr. It has been established that the Co-Cr-Fe-Ni rich zone is the FCC structured in high entropy alloys of AlCoCrFeNi [133,134,135,136,137]. This is also consistent with Butler et al.’s [119] interpretation of oxidized Al_20_Co_25_Cr_25_Ni_25_Si_5_. Contrary to this, the NiAl-rich B2 phase is distinguished by its darker dendritic regions within the substrate [114,128,129]. The similar procedure produces continuous Cr_2_O_3_ production in the A_l9.1_Co_18.2_Cr_18.2_Fe_18.2_Ni_36.3_ sample, coupled with a CoCrFeNi spinel structure at the scale surface. In contrast, non-continuous Al_2_O_3_ precipitates exhibit significant growth depths within the metallic substrate. Also found close to the scale/substrate interface were some Cr_2_O_3_ precipitates. The scales grown on A_l9.1_Co_18.2_Cr_18.2_Fe_18.2_Ni_36.3_ were not enough to address this issue, leading to a persistent mass increase after a certain amount of oxidation time. The majority of results indicate that oxide scales formed on Al_25_Co_25_Cr_25_Ni_25_ and Al_20_Co_25_Cr_25_Ni_25_Si_5_ were adequate for shielding those metallic substrates from notable internal oxidation [132]. This suggests that maintaining a continuous production of Al_2_O_3_ is essential to establish a robust barrier against internal oxidation when operating for extended periods in high-temperature conditions. Al_20_Co_25_Cr_25_Ni_25_Si_5_ appears to have a higher Al contribution, which explains why this material has slightly superior oxidation kinetics [132].

The research of Gawel et al. [132]. found that an AlCoCrFeNi HEA containing aluminum content of less than 9 at.% Al does not always ensure that Al_2_O_3_ layers continue to form inside the scale that forms as the substrate oxidizes. Further, the oxide scale forms a continuous layer made entirely of Cr_2_O_3_ and a mixed/complex oxide phase near the oxidation region. In contrast, it is found that when Aluminum composition reaches a minimum of 20 atomic %, in the case of Al_25_Co_25_Cr_25_Ni_25_ and Al_20_Co_25_Cr_25_Ni_25_Si_5_ materials, a highly protective continuous Al_2_O_3_ layer along with Cr_2_O_3_ is found. Interestingly, oxide materials, such as Cu_2_O, exhibit unique properties that can significantly enhance their functionality [138,139,140]. Whan coupled with HEAs these characteristics make them effective in forming protective layers, contributing to improved applications and material performance.

## 10. Concluding Remarks

In conclusion, this review comprehensively examines the advancements in Ni-containing high-entropy alloys, spanning from fundamental understanding to alloy design intricacies and manufacturing processes. Discussions encompass mechanical properties, additive manufacturing defects, and the influence of factors like valence electron concentration (VEC) and pressure-induced phase transitions. The review also discusses new uses, like replacing TRIP and TWIP steel, and the impressive performance of Ni-based HEAs in resisting thermal shock and oxidation. As research in this field progresses, the insights presented here will continue to guide future investigations and innovations in advanced materials science. This paper also aims to systematically summarize the knowledge surrounding Ni-based high-entropy alloys. Various manufacturing techniques such as casting, additive manufacturing, and powder metallurgy are employed in creating high-entropy alloys, each affecting the characteristics of the alloy differently. Furthermore, additive manufacturing stands out as an up-and-coming method for processing high-entropy alloys, offering unique advantages in terms of design flexibility, rapid prototyping, and creating complex geometries. This capability is especially beneficial for Nickel-containing HEAs, where additive manufacturing techniques can facilitate precise control over alloy composition and microstructure, ultimately enhancing the structural properties and performance. Given their exceptional qualities, HEAs have garnered significant interest, offering many options for tailoring properties through different manufacturing processes and alloy compositions. Particularly noteworthy is the excellent mechanical characteristics exhibited by Ni-containing high-entropy alloys at extreme temperatures, enabling them to be well-suited candidates in structural components for aerospace engines. The formation of aluminum oxide (Al_2_O_3_) and chromium oxides (Cr_2_O_3_) layers during oxidation plays a crucial role in reducing the oxidation rate, preserving the material from surface deterioration. In the future, delving deeper into the thermodynamics of phase formation, studying kinetics, exploring various processing methods, and utilizing computer modeling and simulations holds the potential to open up new avenues in alloy research. As research in this field advances, the insights presented here will continue to serve as a guiding beacon for future investigations and innovations in advanced materials science.

## Figures and Tables

**Table 1:** Variation in configurational entropy of mixing with number of components mixed in equimolar ratios [47].

*n*	ΔS_conf_
1	0
2	0.69R
3	1.10R
4	1.39R
5	1.61R
6	1.79R
7	1.95R
8	2.08R
9	2.20R
10	2.30R
11	2.40R
12	2.49R
13	2.57R

**Table 2:** ∆S_conf_ of some commonly used alloys [47].

System	Alloys	∆S_conf_ at Liquid State
Low-Alloy Steel	4340	0.22R low
Stainless Steel	304	0.96R low
	316	1.15R medium
High speed Steel	M2	0.73R low
Mg Alloy	AZ91D	0.35R low
Al Alloy	2024	0.29R low
	7075	0.43R low
Cu-alloy	brass	0.61R low
Ni-Base Superalloy	Inconel 718	1.31 medium
	Hastealloy	1.37 medium
Co-Base Superalloy	Stellite 6	1.13 medium
BMG	Cu_47_Zr_11_Ti_34_Ni_8_	1.17 medium
	Zr_53_Ti_5_Cu_16_Ni_10_Al_16_	1.3 medium

**Table 3:** Major constituent elements found for use in HEAs [2].

Major Metallic Elements	Minor Metallic Elements	Minor Non-Metallic Elements
Li, Be, Mg	Li, Be, Mg	C, B, Si, P
Al, Sc, Ti, V,	Sc, Ti, V, Cr, Fe	S, O, N
Cr, Fe, Co, Ni,	Co, Ni, Cu, Zn,	
Cu, Zn, Y, Zr	Ga, Ge, Sy, Cd	
Nb, Mo, Sm	Ln, Sn, Sb, Y	
Eu, Au, Gd,	Zr, Nb, Mo, Ru	
Tb, Rh, Pb,	Rh, Pb, Bi, Pd,	
Pd, Ag, Hf, Ta	Ag, Hf, Ta, W, Pt,	
W, Pt, Nd	Au, La, Ce,	
	Pr, Nd, Sm, Fu.	
	Gd, Tb	

**Table 6:** Depicts composition, mixing entropy (ΔS_mix_), and alloy densities for alloys: HESA-1 and HESA-2, respectively [121,124].

Samples	Ni	Al	Co	Cr	Fe	Ti	Ta	Mo	W	∆S_mix_ [-R]
HESA-1	40.7	7.8	20.6	12.2	11.5	7.2	-	-	-	1.58
HESA-2	48.6	10.3	17	7.5	9	5.8	0.6	0.8	0.4	1.56

**Table 7:** The Ƴ/Ƴ’ compositions of HESA-1, HESA-2, and some commercial Ni-based superalloys [121].

	Samples	Ni	Al	Co	Cr	Fe	Ti	Ta	Mo	W	Re	Ru
Ƴ	HESA-1	32.3	4.8	24.7	18.2	16.1	3.9	-	-	-	-	-
	HESA-2	40.1	6.2	22.5	12	14.3	2.4	0.5	1.4	0.6	-	-
	CM247LC	64.9	8.7	11	10.5	-	0.7	0.9	0.2	3.1	-	-
	ME15	53.7	3.6	14.8	20.8	0.1	-	0.1	1.3	3.5	21	-
	Rene’ N5	57.8	5.3	12.8	17.9	-	-	0.1	1.8	1.8	25	-
	RR2100	49.1	3.1	26.8	9.5	-	-	0.3	-	3.7	7.5	-
	RR2101	46	3.1	26.7	9.5	-	-	0.3	-	3.6	7.9	2.9
Ƴ	HESA-1	54.4	9.9	13.7	4.3	5.7	12	-	-	-	-	-
	HESA-2	56.2	10.9	12.3	3.8	5.4	9.2	1.2	0.6	0.4	-	-
	CM247LC	69.4	15.5	5.8	3.4	-	1.4	2.3	0.1	2.1	-	-
	ME15	69	17.7	5.3	2.5	0	-	1.9	0.5	2.9	0.2	-
	Rene’ N5	74.5	15.7	4.3	2.6	-	-	1	0.5	1.2	0.2	-
	RR2100	67.1	16.9	8.6	1.4	-	-	2.6	-	2.9	0.5	-
	RR2101	66.1	16.6	8.8	1.4	-	-	2.7	-	3	0.5	0.9

**Table 8:** Designation and nominal chemical composition of samples investigated by Gawel et al. [132].

Designation	Nominal Chemical Composition [at.%]					
	Al	Co	Cr	Fe	Ni	Si
Al_25_Co_25_Cr_25_Ni_25_	25	25	25	0	25	0
Al_20_Co_25_Cr_25_Ni_25_Si_5_	20	25	25	0	25	5
Al_9.1_Co_18.2_Cr_18.2_Fe_18.2_Ni_36.3_	9.1	18.2	18.2	18.2	36.3	0

**Table 9:** Chemical compositions discovered using EDS point analysis on Al_25_Co_25_Cr_25_Ni_25_, Al_20_Co_25_Cr_25_Ni_25_Si_5_, and Al_9.1_Co_18.2_Cr_18.2_Fe_18.2_Ni_36.3_ cross-sections following oxidation under thermal shock conditions at 1273 K in ambient conditions [132].

Sample	Point	Element [at.%]						
		Al	Co	Cr	Fe	Ni	Si	O
Al_25_Co_25_Cr_25_Ni_25_ (Figure 16a)	1	0.6	1.2	55.4	0.0	1.4	0.0	41.4
	2	35.2	3.5	7.3	0.0	5.3	0.0	43.7
	3	20.3	13.7	19.3	0.0	11.0	0.0	35.2
	4	36.7	1.0	1.4	0.0	0.0	0.0	10.9
	5	5.0	38.2	30.4	0.0	20.3	0.0	5.6
	6	26.2	22.2	8.1	0.0	41.3	0.0	2.2
Al_20_Co_25_Cr_25_Ni_25_Si_5_ (Figure 16b)	1	2.2	0.9	51.3	0.0	1.5	0.0	44.1
	2	84.7	0.4	1.9	0.0	0.0	0.0	13.0
	3	1.8	26.4	46.3	0.0	9.2	7.8	3.5
	4	98.5	0.0	0.7	0.0	0.2	0.6	0.0
	5	4.2	35.5	23.2	0.0	21.3	5.1	5.7
	6	24.6	22.1	3.9	0.0	37.9	3.3	3.2
A_l9.1_Co_18.2_Cr_18.2_Fe_18.2_Ni_36.3_ (Figure 16d)	1	0.4	0.6	54.5	1.3	1.0	0.0	42.2
	2	1.7	18.6	15.9	19.4	34.6	0.0	9.8
	3	50.3	0.7	4.5	0.3	0.4	0.0	43.3
	4	15.3	1.4	41.3	1.9	1.7	0.0	38.3
	5	99.6	0.0	0.4	0.0	0.0	0.0	0.0
	6	3.0	18.1	11.2	20.1	36.2	0.0	11.4

## Data Availability

This is a review study and it deeply analyze the previously reported data to understand the fundamentals for Nickel-Containing High-Entropy Alloys.
